# Quantitative Outcomes of “Y” Scheduling in an Internal Medicine-Pediatrics Residency Program: First Year Data

**DOI:** 10.7759/cureus.77675

**Published:** 2025-01-19

**Authors:** Allen R Friedland, Himani Divatia, Justin Eldridge, Michael Maguire, Chelsea Hastry, Alex Chua, Justin Nichols, Ashley Anttila, John Donnelly

**Affiliations:** 1 Internal Medicine-Pediatrics, ChristianaCare, Newark, USA; 2 Pediatrics, Nemours Children's Health, Wilmington, USA; 3 Internal Medicine-Pediatrics, University of Virginia School of Medicine, Inova Campus, Falls Church, USA

**Keywords:** ambulatory education, block scheduling, continuity clinic, internal medicine-pediatrics, med-peds, residency education, x + y scheduling

## Abstract

Introduction

Scheduling resident continuity clinic sessions and outpatient education in Internal Medicine-Pediatrics (Med-Peds) residency programs pose challenges. A minority of Med-Peds programs have switched to an “X + Y” schedule (each in weeks) specifically to separate inpatient rotations and other experiences (“X” schedule) from resident continuity clinics and other outpatient longitudinal clinical and educational experiences (“Y” schedule). Outside of focus groups, quantitative data from Med-Peds residents on overall and specific satisfaction with an outpatient “Y” schedule and its specific components have not been published. We analyzed our inception year (2022-2023) Med-Peds residents’ satisfaction after transitioning to a new “Y” block of outpatient education and clinical experiences in an average-sized Med-Peds program.

Methods

In July 2022, the “X + Y” schedule was introduced into both our Med-Peds and Pediatrics residency programs with a "6 + 2" (each in weeks) frequency upon an existing "4 + 2" (each in weeks) frequency for Internal Medicine. All Med-Peds residents (n=18) were electronically surveyed at the end of the academic year on the general “Y” schedule, continuity clinics, other outpatient clinical experiences, academic half days, administrative time, patient management skills, and the ability to make personal appointments to support wellness.

Results

All Med-Peds residents (n=18) were highly satisfied/satisfied with their new “Y” schedule (4.7/5) and 92% (12/13) of the postgraduate year (PGY) 2-4 felt much better/slightly better with this schedule template compared to their previous outpatient education design (4.8/5). Clinical outpatient experiences (including continuity clinic), comfort in roles/responsibilities, interest in primary care, administrative time, desktop management, and the ability to make personal appointments to support wellness were highest rated (≥ 4.0/5.0). The Med-Peds residents’ favorite "Y" session was “Cohort Time”, an Academic Half Day with their Med-Peds “Y” cohort colleagues for group learning and socialization.

Conclusions

Despite its complexities, the introduction of the “X + Y” schedule with “Y” continuity clinics and outpatient education dissociated from inpatient and other rotations in our Med-Peds residency program has been widely accepted and highly rated by residents. There has only been the need for minimal modifications for subsequent years.

## Introduction

The Accreditation Council for Graduate Medical Education (ACGME) has separate accreditation and program requirements for categorical Internal Medicine (IM), categorical Pediatrics (Peds), and Internal Medicine-Pediatrics (Med-Peds) residency programs [1-3]. Since Med-Peds programs are dependent on rotations and curricular elements from their categorical counterparts, changes to categorical program requirements occur first, from which modifications to Med-Peds requirements are based. This lag time, which can be years, makes it more difficult for Med-Peds programs to innovate, constantly toggling between old and new categorical and Med-Peds requirements.

Traditionally, resident continuity clinic sessions in IM, Peds, and Med-Peds were included in inpatient rotations. One survey of five Peds residencies found that these sessions were not satisfying, there were handoff concerns, and dissatisfaction from residents leaving another rotation to go to clinics [4]. With the goal to separate inpatient and other rotations (“X”) from resident continuity clinic and other longitudinal outpatient sessions (“Y”), or “ X + Y” scheduling (each in weeks), the first published data demonstrating this educational redesign to be a positive and an accepted innovation was published in 2010 in IM programs [5].

Since that time, changes to ACGME requirements have allowed novel scheduling for categorical IM programs that have resulted in a proliferation of “X + Y” scheduling [1]. The ACGME, through its Advancing Innovation in Residency Education (AIRE) pilot in 2018-2023, allowed categorical Peds and Med-Peds programs to trial different approaches to continuity clinic scheduling that include “Y” scheduling by granting a waiver from the following core Residency Committee requirement related to continuity clinic scheduling: “The sessions must not be scheduled in fewer than 26 weeks per year" [6]. A 2023 Med-Peds Program Directors Association survey (Unpublished Presentation: Anoop Agrawal. Med-Peds Annual Survey. Med-Peds Program Directors Association Annual Meeting; March 30, 2023), suggested that only 15 of the 79 Med-Peds programs had an “X + Y” schedule by themselves, with IM, or both categorical programs. The annual ACGME Resident Survey for Med-Peds residents does not specify the curricular elements that may need improvement like outpatient training or continuity clinic [7]. However, based on our yearly electronic surveys of the Med-Peds residents, we found that our traditional combined continuity clinic and its preclinic conference (imbedded in inpatient and other rotations) to be rated as 3.0/5.0 and 2.4/5.0, respectively (Unpublished Survey: Allen Friedland. Resident Survey of Program; June 2021), and so this was reported to our Program Evaluation Committee (PEC) as a programmatic improvement goal in our Med-Peds Annual Program Evaluation (APE). This was a large motivator to apply to join the AIRE pilot in 2021.

Different constructs of “X + Y” scheduling in Med-Peds programs have been published recently by a group of Med-Peds Program Directors [8], along with focus group data on the pros and cons of this new scheduling model in Med-Peds [9]. However, anonymous survey data on resident satisfaction with the “Y” schedule and its specific curricular components in Med-Peds have not been published. We present data from the first year of experience with the new “Y” schedule for outpatient education, continuity clinic, and longitudinal outpatient experiences.

## Materials and methods

This survey study of residents was conducted at ChristianaCare, Newark, Delaware, United States. The Christiana Care Institutional Review Board (IRB) reviewed the protocol and determined the study to be exempt from IRB oversight per exempt category 45 CFR 46.104(d)(4)(ii) (approval number: #605144). All Med-Peds residents (n=18) were included in the survey and completed an electronic survey at the end of the 2022-2023 academic year. Of the 18 Med-Peds participants, five were from postgraduate year (PGY) 1, five were from PGY 2, four were from PGY 3, and four were from PGY 4.

Background

In 2022, our categorical Peds and Med-Peds programs were accepted into Cohort Five of the ACGME AIRE pilot, granting us a waiver from the following core Residency Committee requirement related to continuity clinic scheduling: “The sessions must not be scheduled in fewer than 26 weeks per year” [2,3]. 

Prior to “X + Y” scheduling, the Med-Peds combined continuity clinic sessions occurred on electives and non-ICU inpatient rotations with a preclinic conference at the beginning or end of the session with separate Med-Peds Academic Half Day (AHD) sessions scattered during the year when the most residents were available. Planning “X + Y” scheduling started in July 2021 and was implemented in July 2022. Both categorical Peds and Med-Peds programs, initiated at the same time an “X + Y” with a “6 + 2” (each in weeks) frequency (or eight weeks for each cycle) upon an existing categorical IM program frequency of “4 + 2" (each in weeks). All programs worked together to prepare and plan for the transition.

With the change to “6 + 2” scheduling, all 18 Med-Peds residents (transitioning to an increase in complement to 20 Med-Peds residents by 2024) were divided into four cohorts of four or five residents, based on schedule requests but each cohort included one or more members from each resident PGY class. Each cohort had six “Y” blocks over the academic year with four selected weeks without any “Y” blocks to allow each cohort to have the same amount of “Y” time each academic year (Table 1).

**Table 1:** Representative Med-Peds yearly "X + Y" schedule The Med-Peds residents were split into four cohorts for the academic year with residents from each PGY class; each column equals two weeks in duration and each "Y" equals two weeks; for rows, each unlabelled field equals two weeks in duration and is "X" weeks. Only Cohort 1 is completely filled for illustration purposes

	1	2	3	4	5	6	7	8	9	10	11	12	13	14	15	16	17	18	19	20	21	22	23	24	25	26
Cohort 1	Y1	X	X	X	Y2	X	X	X	Y3	X	X	X	No Y	Y4	X	X	X	Y	X	X	X	Y6	X	X	X	No Y
Cohort 2		Y1				Y2				Y3			No Y		Y4				Y5				Y6			No Y
Cohort 3			Y1				Y2				Y3		No Y			Y4				Y5				Y6		No Y
Cohort 4				Y1				Y2				Y3	No Y				Y4				Y5				Y6	No Y

Med-Peds PGY 1-3 residents had a combined continuity clinic at the same time and practice location. Med-Peds PGY 4 residents had a different combined continuity clinic location with different days for their continuity sessions from the rest of their cohort, allowing the PGY 3 residents to be seniors of their “Y” cohort, decrease competition for other clinical “Y” experiences, and offer different clinical opportunities that were available on other days of the week. All AHD sessions (IM, Peds, and Med-Peds) included the corresponding Med-Peds cohort.

Each resident was scheduled for 48 continuity sessions for the year with the goal to maintain at least 40 sessions, a 10%+ increase from the 36 sessions prior to “X + Y”, allowing for cancellations due to retreats, certifications, and a myriad of other reasons. Longitudinal clinical outpatient experiences (not continuity clinic) were scheduled differently across all four resident years where possible, to provide graded autonomy and personalization: (i) PGY 1+2: Clinical experiences focused more on foundational direct patient care experiences such as general pediatrics and general medicine in different settings, and (ii) PGY 3+4: Clinical experiences focused more on advanced clinical experiences with career personalization such as gender health, special health care needs, and precepting.

Each Med-Peds cohort participated in the corresponding Peds and IM AHDs during their "Y" blocks with the categorical residents that included basic topics in outpatient care, patient management skills in quality improvement (QI), and population health. The Med-Peds program designed its own AHD, named “Cohort Time” that was self-directed and designed by each cohort to include academics and wellness activities in a setting of their choice on or off campus. Administrative time was added in the second half of the academic year and became a permanent feature to allow for desktop management, module completion, certifications, and personal appointments to be scheduled and completed.

A representative schedule of one week for PGY 1-3 (repeated in week 2) of the “Y” schedule is shown in Table 2.

**Table 2:** Representative Med-Peds weekly "Y" schedule CE: clinical experience; CP: continuity practice; AHD: Academic Half Day; Peds: pediatrics; IM: internal medicine; Med-Peds: internal medicine-pediatrics; PGY: postgraduate year PGY 1+2: CE focuses more on foundational direct patient care experiences; PGY 3+4: CE focuses more on advanced clinical experiences with career personalization.

	Monday	Tuesday	Wednesday	Thursday	Friday
AM	CE	CE or Administrative time	AHD- Peds	AHD- IM	CP
PM	CE	CP	CP	AHD- Med-Peds or "Cohort Time"	CP

Data collection

SurveyMonkey (SurveyMonkey Inc., San Mateo, California, United States) was used to create a questionnaire (See Appendices) and collect electronic anonymous data at the end of the academic year from the Med-Peds residency program and not from any other AIRE participating program. Questions were scored on a Likert scale of 1 - 5 where 1=highly dissatisfied/much worse, 2=dissatisfied/slightly worse, 3=neutral/about the same, 4=satisfied/slightly better, 5=highly satisfied/much better. The survey for the Med-Peds residents (n=18) focused on the following areas: general "Y", continuity clinic, other clinical experiences, academic half days, administrative, patient management skills, and wellness. A total of 13 residents (which excluded the five in PGY 1) answered a question to self-compare between the new and old schedule for outpatient education. This was because the five residents in PGY 1 could not compare back to the previous traditional schedule template. For all other questions, except where noted in the Results section, all 18 residents completed all survey questions.

Data analysis

Historical baseline data was obtained from the residency program's global electronic annual survey that examined all aspects of the program but did ask one question each in general about their continuity clinic and preclinic conference experience using a five-point Likert scale. Baseline data compared to implementation data was statistically analyzed using an independent t-test. P<0.05 was considered significant using SAS 9.4 (SAS Institute Inc., Cary, North Carolina, United States) for the analysis.

## Results

General “Y”

All Med-Peds residents (n=18) were highly satisfied/satisfied (4.7/5.0) with their new “Y” schedule. Of the 13 PGY 2-4, 12 (92%) felt much better/slightly better (4.6/5.0) with this schedule template compared to their previous outpatient education design. No resident felt that this new “Y” design was worse than their previous model. In addition, 17 (94%) residents felt highly satisfied/satisfied with the comfort they had in their roles and responsibilities (4.7/5.0) and with their balance of independence and group work (4.4/5.0).

Regarding residents’ interest in primary care, 14/17 (82%) rated it much better/better after one year of the “Y” schedule, and 3/17 (18%) residents were neutral (4.4/5.0).

Other clinical experiences and continuity clinics

Resident satisfaction in greater than 30 clinical outpatient experiences excluding continuity clinic had a cumulative average of 4.5/5.0 with a range of 3.0-5.0. Most residents (14/16, 88%) were highly satisfied/satisfied with their own continuity clinic sessions (4.4/5.0) which was significantly improved (p < 0.001) compared to historical data (3.0/5.0) prior to the "Y" schedule. The vast majority of residents (12/13 or 92%) were highly satisfied/satisfied with the continuity of seeing their own patients or their Med-Peds cohorts’ patients (4.8/5.0).

AHD

AHD in Peds and IM were similarly rated: 13/18 (78%) residents were highly satisfied/satisfied (3.9/5.0). AHD in Med-Peds (“Cohort Time”) was most highly rated by residents; 18/18 (100%) were highly satisfied/satisfied (4.8/5.0) which was significantly improved compared to historical data (2.4/5.0) prior to the "Y" schedule (p < 0.001).

Administrative time, patient management skills, wellness

**Figure 1:**
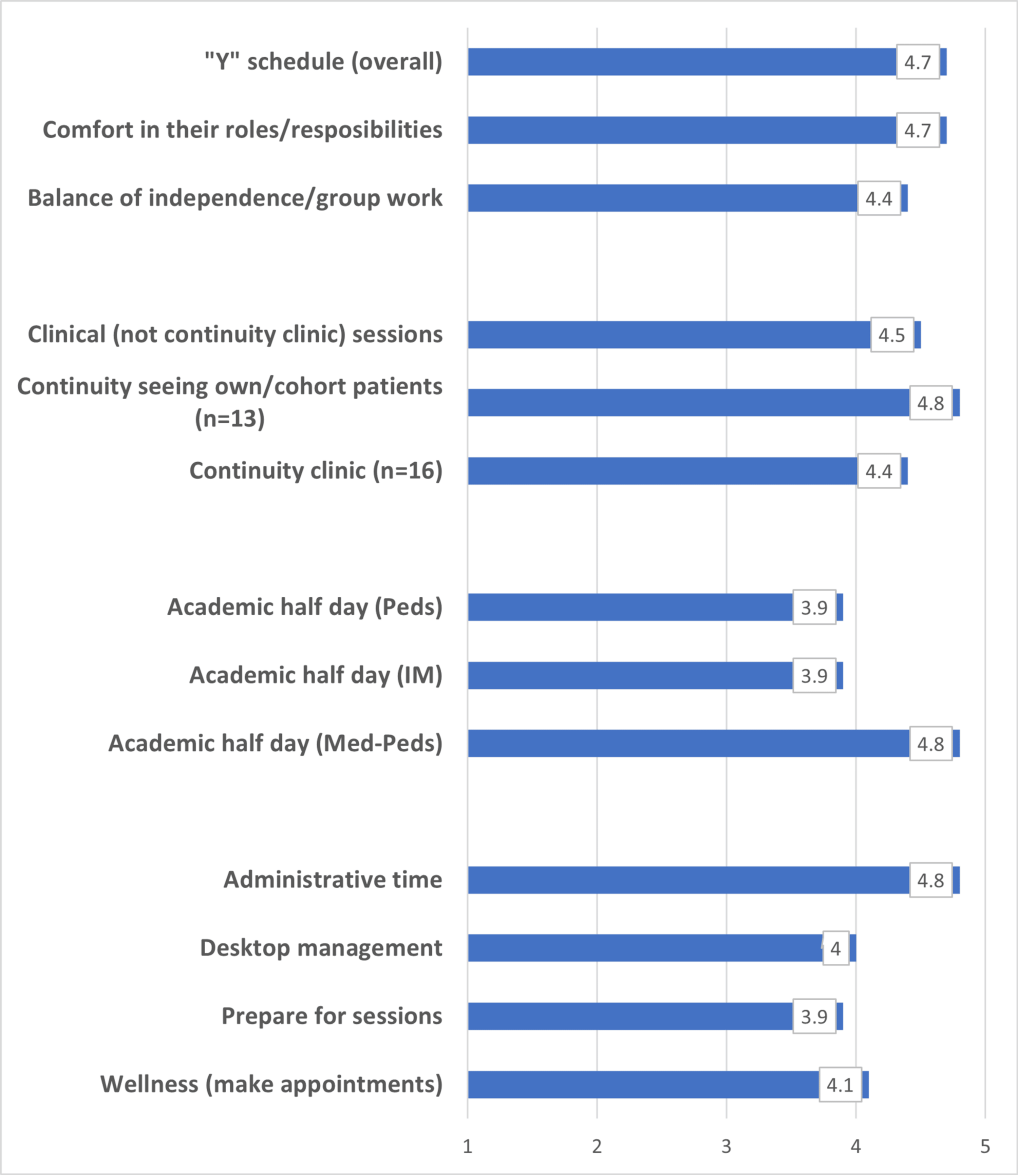
Resident satisfaction with "Y" schedule elements Item responses options are 1 = highly dissatisfied, 2 = dissatisfied, 3 = neutral, 4 = satisfied, 5 = highly satisfied N=18 unless otherwise stated. Peds: pediatrics; IM: internal medicine; Med-Peds: internal medicine-pediatrics

**Figure 2:**
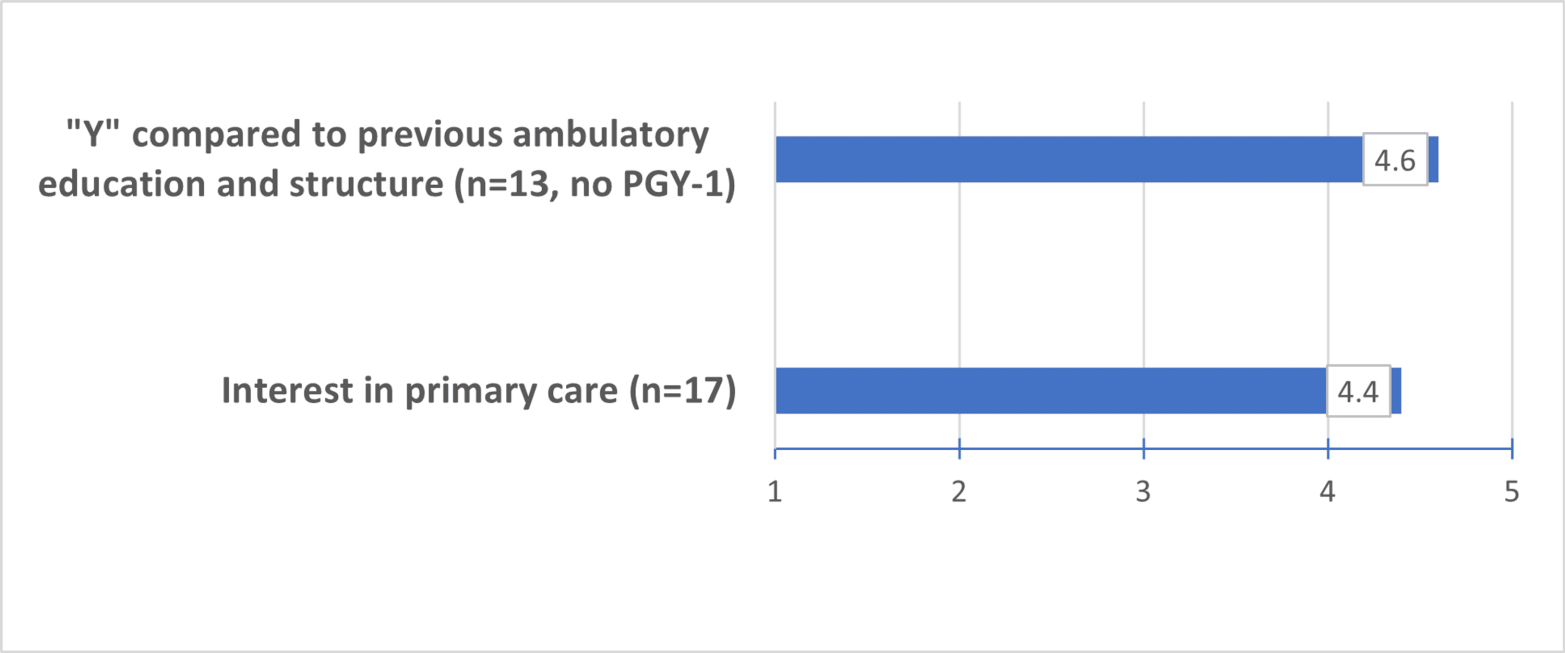
Comparison of current ambulatory education/structure to previous structure and primary care interest Item responses options are 1 = much worse, 2 = worse, 3 = about the same, 4 = slight better, 5 = much better PGY: postgraduate year

All residents were highly satisfied/satisfied (4.8/5.0) with the administrative time provided. Patient management skills using desktop management (4.0/5.0) and the ability to prepare for sessions (3.9/5.0) were similarly rated. The ability to make personal appointments supporting wellness was rated as follows: 13/18 residents (72%) were highly satisfied/satisfied, 4/18 (22%) were neutral, and 1/18 (6%) were dissatisfied. The overall rating was 4.1/5.0. See Figures 1 and Figure 2 for a summary of results.

## Discussion

To date, this is the only reported Med-Peds residency program’s quantitative data on the “Y” outpatient schedule and its specific components. The study findings are important as ACGME draft requirements in Med-Peds are scheduled to be approved in February 2025 and are predicted to reduce barriers for more programs to develop and use this system of scheduling outpatient training and education [10]. Our model can also be used as a standard for other newly ACGME-accredited combined residency program types that have a continuity clinic [11], along with other ACGME specialties that are starting to heighten their focus on outpatient training and education [12,13]. It is difficult to compare our data with data reported in a systematic review that included 11 studies in IM with an “X + Y” schedule [14] and in the first published report of five pediatric “X + Y” programs [15], since these studies, outside of overall satisfaction, had little to no overlap to our areas of study and focus, which included many new and specific education and clinical components to our "Y" schedule.

The inception year of our outpatient education and schedule demonstrated success with our goal to provide satisfying experiences using an outpatient focus of training for the Med-Peds residents. In both areas where there were pre-implementation data and ratings of Med-Peds-specific outpatient education (preclinic conference compared to AHD) and continuity clnics, statistical significance was reached. The switch to an “X + Y” schedule aligns us much closer with the schedule used by our categorical Peds and IM programs and has been the largest schedule and curricular change to our program to date.

As the only Med-Peds publication with “Y” quantitative data, our findings support the highest satisfaction (≥ 4.0 or highly satisfied/satisfied) in the following (i) clinical areas: continuity clinic (including seeing own/cohort patients), outpatient clinical experiences, comfort in roles/responsibilities and interest in primary care, (ii) educational sessions: Med-Peds AHD, and (iii) administrative areas: administrative time, balance of independence/group work, ability to make appointments to support wellness, and desktop management. Areas of lesser satisfaction (<4.0 or satisfied/neutral) include both AHD in Peds and IM and the ability to prepare for sessions. No component was rated below a 3.9/5.0, and so it can be assumed that all areas studied were successful.

Reasons for higher satisfaction areas (≥ 4.0) may relate to the new introduction of longitudinal clinical experiences, first-day orientation in the first cycle for each cohort, open dates for continuity clinic for the entire year in advance, more continuity sessions in the year, and the ability for staff/residents to plan future visits by patients for the residents/cohorts. Reasons for lesser satisfaction areas (<4.0) may relate to travel time between AM and PM experiences, and an increased number of continuity sessions making it more difficult to fully prepare charts for clinical sessions. 

Each cohort of Med-Peds residents participated in all three AHDs to allow for identity formation for each Med-Peds cohort and also to allow educational integration with both categorical programs. Ratings for IM AHD and Peds AHD were rated the same. The highest-rated educational session, Cohort Time (Med-Peds AHD), was expected since Med-Peds residents were given the freedom to choose the setting and content of these group learning and social sessions based on the needs of their own cohort. The self-reported increase in interest in primary care will need further study to determine if there is an actual increase in interest in primary care careers, a better appreciation of what primary care provides, or a better schedule and approach to primary care training.

It is difficult to conclude the impact of the “X + Y” schedule on actual resident wellness since it is such a complicated term to define and measure, especially without baseline data, but we believe better promotion of the flexibility for residents to pursue personal appointments is important.

The same survey as in the present survey was sent to all residents at the end of the next academic year and 100% were highly satisfied/satisfied with the Y schedule (4.8/5.0) (not published or fully analyzed) and the results appear remarkably similar to year one data except for a few specialty clinics that needed improvements or were changed. However, with each year after implementation, it will become more difficult to study the “Y” schedule impact as more residents would not have experienced any other schedule type.

Study limitations

The data are subjective, observational in nature, and without robust pre-implementation data included and thus causation from the present intervention cannot be proven. There are no data from the categorical programs to allow for comparison or views about their own "Y" schedule, AHD, and the impact of the schedule changes on the categorical residents. There was no measurement from the patient's perspective or continuity reports. Validated wellness scales were also not provided to residents to get a real sense of changes over the academic year.

A robust faculty survey would need to be developed to capture data on the perceptions of faculty with the “X + Y” schedule. Measurements of the impact of our changes on our categorical residents could also provide unique insights. Based on local needs and interests, each Med-Peds program will require modifications to this model which may make it difficult to compare. This study does not consider the effect of new ACGME Med-Peds requirements and its impact on the flexibility of the "Y" schedule. Our residency program size, location, learner needs, and administrative support limits generalizability.

Future research

Since the majority of Med-Peds programs may soon have the opportunity to transition to an "X + Y" schedule, the Med-Peds community would benefit from a validated pre- and post-implementation survey that includes the categorical programs. Also, a faculty (preceptor) and practice survey (staff, patients) including metrics to see if there is an impact of this approach on the preceptors' ability to teach, staff interaction with the residents, continuity of care, and patient satisfaction would be beneficial. Postgraduate surveys may also give insight into whether this model has been helpful.

## Conclusions

There was a need to change our Med-Peds program's approach to outpatient education and training. With extensive stakeholder engagement and planning for the entirety of complexities, a new outpatient structure using a “Y” schedule approach for Med-Peds continuity clinic, other outpatient clinical and learning experiences can be successfully instituted with broad resident acceptance and high satisfaction ratings. The Med-Peds AHD (Cohort Time) is the most highly rated academic session. Only minimal adjustments were needed based on this study for subsequent years of this innovative Med-Peds schedule and built on the small literature specific to Med-Peds programs. Future studies should have pre- and post-surveys that focus on patients’ continuity and outcomes, faculty and other staff member satisfaction, and the impacts on all resident groups that participate in “Y” scheduling in conjunction with a Med-Peds program.

## Appendices

**Table 3:** "Y" Survey

Please rate your overall satisfaction for the entire year.					
	Highly Dissatisfied	Dissatisfied	Neutral	Satisfied	Highly Satisfied
“Y” Schedule					
Please express the level of satisfaction with the clinical opportunities you have participated for the entire year.					
	Highly Dissatisfied	Dissatisfied	Neutral	Satisfied	Highly Satisfied
Continuity Sessions					
Continuity with your patients/cohort patients					
Peds Practice Program- Christiana					
Green Module- Nemours					
EPIC- YMCA					
HIV- Peds					
HIV- Adult					
Women’s Health					
Urology					
Weight Management- ChristianaCare					
Weight Management- Nemours					
Center for Special Health Care Needs- Primary Care					
Center for Special Health Care Needs- Primary Care Sickle Cell					
Center for Special Health Care Needs- Specialty Care Sickle Cell					
Center for Special Health Care Needs- Cystic Fibrosis					
Center for Special Health Care Needs- Down Syndrome					
Center for Special Health Care Needs- Hemophilia					
Center for Special Health Care Needs- Cerebral palsy, GI or urology					
Transition Program- Nemours					
Pediatric Down Syndrome- Nemours					
Westside HealthCare					
Warner School					
First State School					
Center for Virtual Health					
Sports Medicine					
Community Health Workers					
Hope Center					
Addiction Medicine					
Med-Peds Service					
Personalized experiences for you					
Other					
Please express the level of satisfaction with the non-clinical opportunities you have participated for the entire year.					
	Highly Dissatisfied	Dissatisfied	Neutral	Satisfied	Highly Satisfied
Peds- Academic Half Day					
Internal Medicine- Academic Half Day					
Med-Peds- Academic Half Day (Cohort Time)					
Administrative time					
Wellness and ability to make appointments etc.					
Desktop management					
Ability to prepare for sessions					
Balance of independence/group work (Teamwork)					
I am more comfortable with my roles and responsibilities					
Compared to previous ambulatory education, our new “Y” education and structure is (No PGY-1)					
	Much Worse	Slightly Worse	About the Same	Slightly Better	Much Better
Compared to our previous ambulatory education, our new “Y” education and structure is					
Interest in Primary Care					
